# Membrane Toxicity of Abnormal Prion Protein in Adrenal Chromaffin Cells of Scrapie Infected Sheep

**DOI:** 10.1371/journal.pone.0058620

**Published:** 2013-03-04

**Authors:** Gillian McGovern, Martin Jeffrey

**Affiliations:** Animal Health and Veterinary Laboratories Agency, Lasswade Laboratory, Pentlands Science Park, Penicuik, Midlothian, Scotland; Dulbecco Telethon Institute and Mario Negri Institute for Pharmacological Research, Italy

## Abstract

Transmissible spongiform encephalopathies (TSEs) or prion diseases are associated with accumulations of disease specific PrP (PrP^d^) in the central nervous system (CNS) and often the lymphoreticular system (LRS). Accumulations have additionally been recorded in other tissues including the peripheral nervous system and adrenal gland. Here we investigate the effect of sheep scrapie on the morphology and the accumulation of PrP^d^ in the adrenal medulla of scrapie affected sheep using light and electron microscopy. Using immunogold electron microscopy, non-fibrillar forms of PrP^d^ were shown to accumulate mainly in association with chromaffin cells, occasional nerve endings and macrophages. PrP^d^ accumulation was associated with distinctive membrane changes of chromaffin cells including increased electron density, abnormal linearity and invaginations. Internalisation of PrP^d^ from the chromaffin cell plasma membrane occurred in association with granule recycling following hormone exocytosis. PrP^d^ accumulation and internalisation from membranes is similarly associated with perturbations of membrane structure and trafficking in CNS neurons and tingible body macrophages of the LRS. These data suggest that a major toxic effect of PrP^d^ is at the level of plasma membranes. However, the precise nature of PrP^d^-membrane toxicity is tissue and cell specific suggesting that the normal protein may act as a multi-functional scaffolding molecule. We further suggest that the co-localisation of PrP^d^ with exocytic granules of the hormone trafficking system may provide an additional source of infectivity in blood.

## Introduction

Transmissible spongiform encephalopathies (TSEs) or prion diseases are chronic, fatal, neurodegenerative diseases affecting humans and animals which may be acquired following oral exposure to infectivity. Bovine spongiform encephalopathy (BSE) and variant Creutzfeldt - Jakob disease (vCJD) occur as a result of the consumption of BSE contaminated offal [1], [2], kuru is associated with cannibalistic rituals [3] and chronic wasting disease and scrapie are also thought to be acquired orally through infected pastures or the environment [4], [5].

The presence of TSE infectivity in both brain and lymphoid tissue is usually associated with the accumulation of disease specific forms of the normal cellular form of the prion protein molecule (PrP^c^). PrP^c^ is expressed abundantly within the central nervous system (CNS) [6], [7] but also within many other tissues including those of the lymphoreticular system (LRS), and the adrenal medulla and pituitary glands of the endocrine system [6], [8], [9].

Immunohistochemically detected disease specific forms of PrP (PrP^d^) accumulate in the CNS, the LRS and peripheral nervous system in most naturally infected and experimental animal TSEs and in human vCJD. In the CNS, PrP^d^ can be identified in association with glial cells and neurons in sheep, cattle, mice and man [10]–[13] where it co-localises with abnormal membrane microfolding and invaginations [12], [14]. Within the LRS, tingible body macrophages and follicular dendritic cells accumulate PrP^d^ [15]. Tortuous dendritic extensions of FDCs are intricately linked with plasmalemmal PrP^d^ accumulation [16].

Variant CJD, naturally occurring animal TSEs and some experimental rodent models of scrapie, also show infectivity and PrP^d^ accumulation in Peyer’s patches of intestine, enteric nervous system and other parts of the autonomic peripheral nervous system. Studies of the sequence of tissue PrP^d^ accumulation in experimental rodent [17] and hamster scrapie [18], [19], natural sheep scrapie [5], [20] and experimental cattle [21] and sheep BSE [22] indicate more specifically that sympathetic noradrenergic fibres are likely to be responsible for transport of infectivity to the intermediolateral column (IML) of the caudal thoracic spinal cord and via parasympathetic nerve fibres of the vagus nerve to the hindbrain medulla.

**Figure 1:**
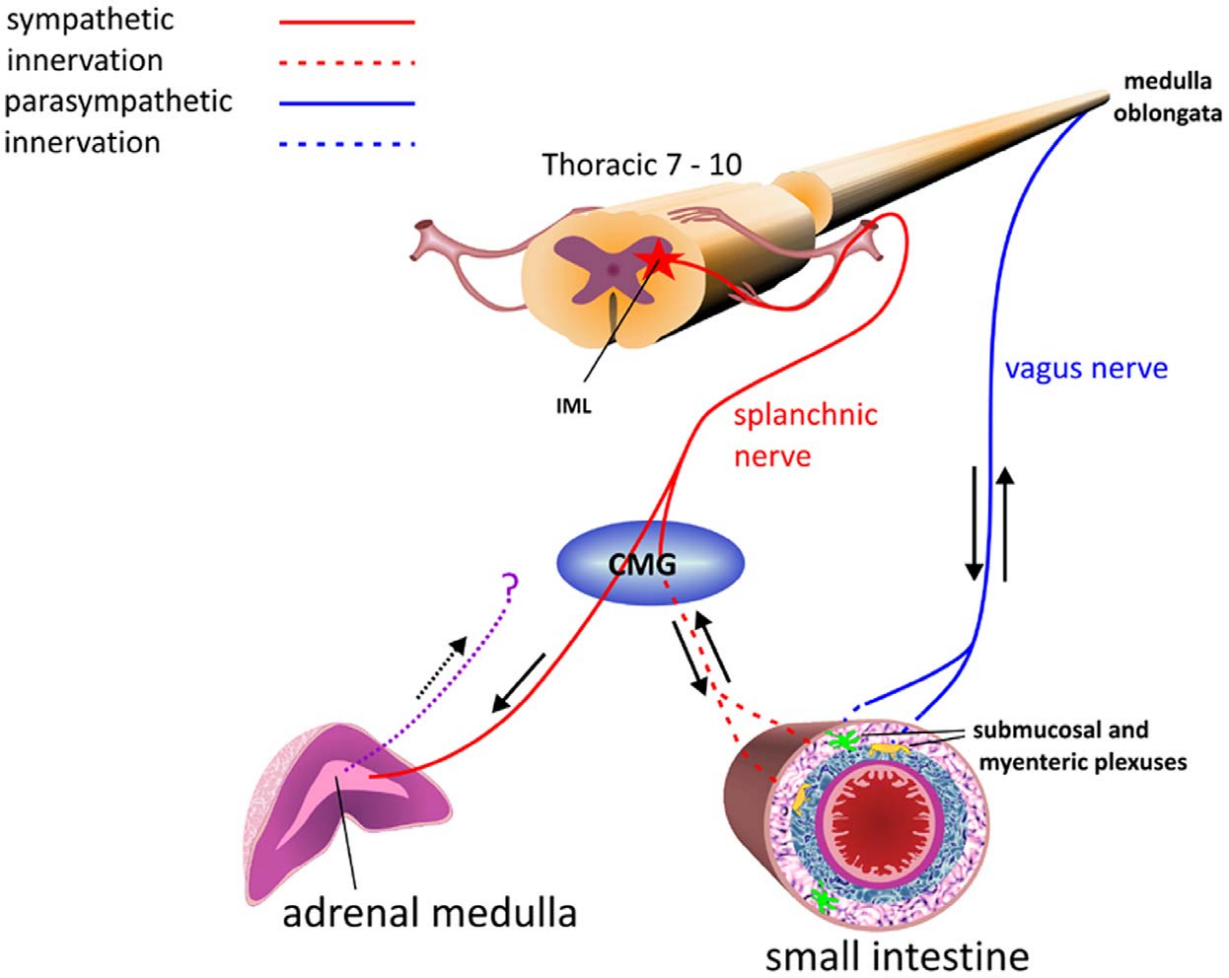
Diagram showing routes of innervation of the small intestine and adrenal medulla. Parasympathetic signals travel between the medulla oblongata and the small intestine via the vagus nerve while sympathetic signals are received and transmitted following synapse of the splanchnic nerve within the celiac mesenteric ganglion (CMG) The adrenal medulla receives acetylcholine stimulation directly from the splanchnic nerve following signals from the intermediolateral column (IML) of the spinal cord. Ascending axons may also transmit retrograde signals from the adrenal medulla.

The principal cell of the adrenal medulla is the hormone producing chromaffin cell. These cells are modified postganglionic cells, originating within the neural crest which, during foetal development, lose their axons and dendrites. They continue to receive innervation from corresponding preganglionic fibres of the sympathetic nervous system. The adrenal medulla receives both sympathetic motor and sensory innervation [23], allowing sympathetic, autonomic control of the synthesis of noradrenaline (NA) and adrenaline (A) from chromaffin cells. The adrenal medulla is composed of approximately 20 per cent NA cells and 80 per cent A cells which, following stimulation by acetylcholine released from preganglionic sympathetic fibres, release hormones into the extracellular space. Endothelial cells of the capillaries represent the barrier through which hormones must pass in order to reach the blood [24], [25]. Preganglionic sympathetic fibres of the splanchnic nerve pass from the IML of the thoracic spinal cord directly to the adrenal medulla via the celiac mesenteric ganglion (CMG) (figure 1). Recent studies have demonstrated that ganglion cells from within the adrenal medulla are also likely to project ascending axons and may relay information from the adrenal medulla to the CNS [26], [27]. The nature of the immunolabelling pattern of the adrenal medulla of BSE infected cattle [28] and scrapie infected sheep [29] indicate that fine preganglionic sympathetic nerve processes may accumulate PrP^d^. There remain two possible routes by which the adrenal medulla may become infected following natural infection. Infection may occur via the blood, ascending to the CNS, or infection may reach the CNS from the gut and is then disseminated to the adrenal medulla via the splanchnic nerve (figure 1).

In the current study, we aimed to determine the subcellular location of PrP^d^ in the adrenal medulla, and morphological responses of cells to PrP^d^ accumulation. Our study shows that chromaffin cells are the primary location of PrP^d^ accumulation within the adrenal medulla, with only occasional involvement of nerve terminals. We further show that the PrP^d^ accumulates in association with morphologically altered plasma-membranes.

## Materials and Methods

### Animals and Experimental Procedure

All experiments involving animals carried out within AHVLA are supervised by a named Veterinary surgeon as required under UK legislation and individual experiments are approved by UK government Home office inspectors. The study of animals within this experiment has been approved by Defra.

Five sheep naturally (n = 3) or subcutaneously (n = 2) infected and with clinical signs of scrapie, and 2 uninfected control animals were euthanized and adrenal gland removed. All scrapie infected sheep were taken from susceptible PrP genotypes ARQ/RQ^136^ (n = 3), VRQ/VRQ^136^ (n = 1) and VRQ/ARQ^136^ (n = 1) where the letters A, R, Q, and V represent respectively the single letter codes for amino-acids alanine, arginine, glutamine and valine.

Left adrenal glands were halved and immersion fixed in buffered formal saline or 4% paraformaldehyde. One millimetre thick slices of adrenal cortex and medulla were taken for paraffin wax embedding and slices of adrenal medulla for electron microscopy. Tissues selected for electron microscopy were further trimmed into 1 mm^3^ blocks, post fixed in osmium tetroxide and embedded in araldite.

#### Light microscopy procedure – wax

The light microscopical immunohistochemical procedure was used as described previously [10] using anti-PrP antibody R523.7 specific to the C terminus of the PrP molecule (J. Langeveld, ID–Lelystad, Netherlands). This was applied over-night at 27°C, at a dilution of 1∶12000 in incubation buffer. Protease K resistance of PrP was not tested, therefore the term PrP^d^ will be used to indicate all disease-specific PrP accumulations. Double labelling using 523.7 and synaptophysin was carried out based on the technique described by Siso et al. [30] in order to determine whether PrP^d^ co-localised with synapses. Synaptophysin was applied overnight at a 1∶400 dilution and visualised using vector VIP kit.

#### Light microscopy procedure – resin

As described previously [31], the avidin-biotin complex immunohistochemical staining method was applied to the etched and pre-treated sections using R523.7. Substantial labelling was present within tissues embedded in resin from scrapie-affected animals. Selected blocks with appropriate immunolabelled areas and control blocks containing adrenal medulla were then taken for sub-cellular studies. Again, the antibody used does not distinguish between the protease-sensitive and protease-resistant isoforms of PrP in biochemical extracts, however, the method employed in the study of TSE pathology in resin embedded tissues does not show any PrP labelling in control tissues. PrP detected in clinically affected sheep is therefore by definition, disease associated. Multiple sections from at least 2 blocks containing adrenal medulla were studied from each animal.

#### Ultrastructural immunohistochemical procedure

65 nm sections were taken from resin blocks previously found to show PrP^d^ labelling (or controls containing adrenal medulla) and immunolabelled as described previously [31]. Sheep PrP^d^ was detected using primary antibody R523.7 at a 1∶250 dilution in incubation buffer. A pre-immune serum was used as a control. Between 3 and 6 1 mm^2^ sections were studied from all available animals. To estimate the extent of PrP^d^ accumulation and membrane malformation of chromaffin cells from scrapie infected animals, we studied all micrographs taken (300+), scoring membranous immunogold deposit and abnormal electron dense or “zig-zag” membranes.

## Results

### Light Microscopical Analysis of Adrenal Medulla

**Figure 2:**
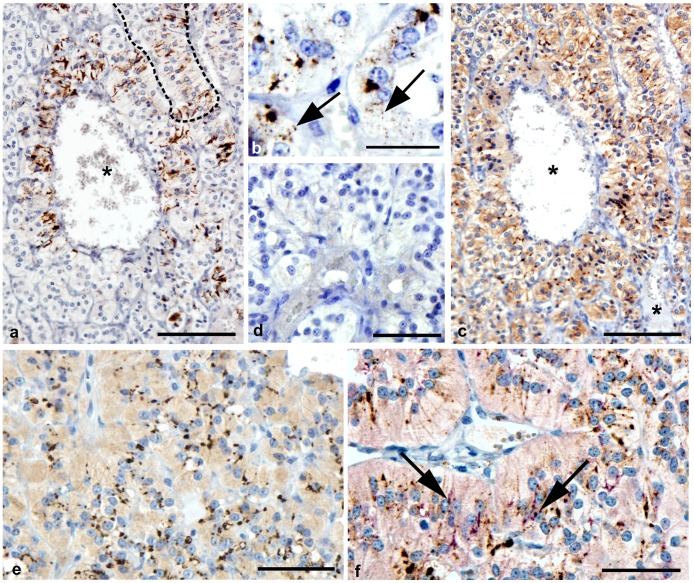
Detection of PrP^d^ and synaptophysin in adrenal medulla of normal and scrapie-affected sheep. A: Bar = 100 µm. Paraffin wax embedded adrenal medulla from a scrapie affected sheep. Linear profiles of PrP^d^ immunolabelling appear at the cytoplasmic margins of chromaffin cells which are arranged around a venous sinusoid (asterisk). A cord of chromaffin cells is indicated by the dotted line. B: Bar = 25 µm. Paraffin wax embedded adrenal medulla from a scrapie affected sheep. Granular intracellular PrP^d^ labelling is also associated with chromaffin cells (arrows). C: Bar = 100 µm. Paraffin wax embedded adrenal medulla from a scrapie affected sheep (serial section of that shown in A). Similarly, synaptophysin immunolabelling of synapses forms intense puncta at the cytoplasmic margins of chromaffin cells. Large and small venous sinusoids are labeled (asterisks). D: Bar = 50 µm. Paraffin wax embedded adrenal medulla from a normal sheep. No PrP^d^ immunolabelling is present. E: Bar = 50 µm. Wax embedded adrenal medulla from a normal sheep. Punctate synaptophysin labelling is apparent at the cytoplasmic margins of chromaffin cells. F: Bar = 50 µm. Paraffin wax embedded adrenal medulla from a scrapie affected sheep. Multiple puncta of pink synaptophysin labelling are easily visible, however only sparse co-localisation (purple) (arrows) with the dark brown PrP^d^ labelling occurs.

The adrenal medulla consists of large, closely packed cells which were arranged in clusters or cord like structures. Between these structures were both small and large venous sinusoids (figure 2a and 2c). PrP^d^ labelling was restricted to the medulla of the adrenal gland and was similar in pattern in each scrapie affected sheep although the intensity and distribution varied between animals. Labelling was primarily associated with chromaffin cells which are the predominant cell type of the medulla, and formed linear patterns, apparently between adjacent cells (figure 2a). Of the tissues studied, we estimate that more than 70% of chromaffin cells accumulate PrP^d^. Less frequently, intracellular granules could be observed within the cytoplasm (fig. 2b). No PrP^d^ labelling was observed within medulla of adrenal glands of uninfected sheep (figure 2d).

Using synaptophysin antibody which specifically labels membrane of synaptic vesicles both in the CNS and periphery, punctate granular labelling was observed surrounding cells of the medulla in both the scrapie infected and normal animals (figure 2c and 2e). Double IHC for PrP^d^ and synaptophysin revealed that only a small proportion of PrP^d^ co localised with synapses of peripheral nerves within the medulla of scrapie infected animals (figure 2f).

### Ultrastructural Analysis of the Morphology of Uninfected Sheep Adrenal Medulla

**Figure 3:**
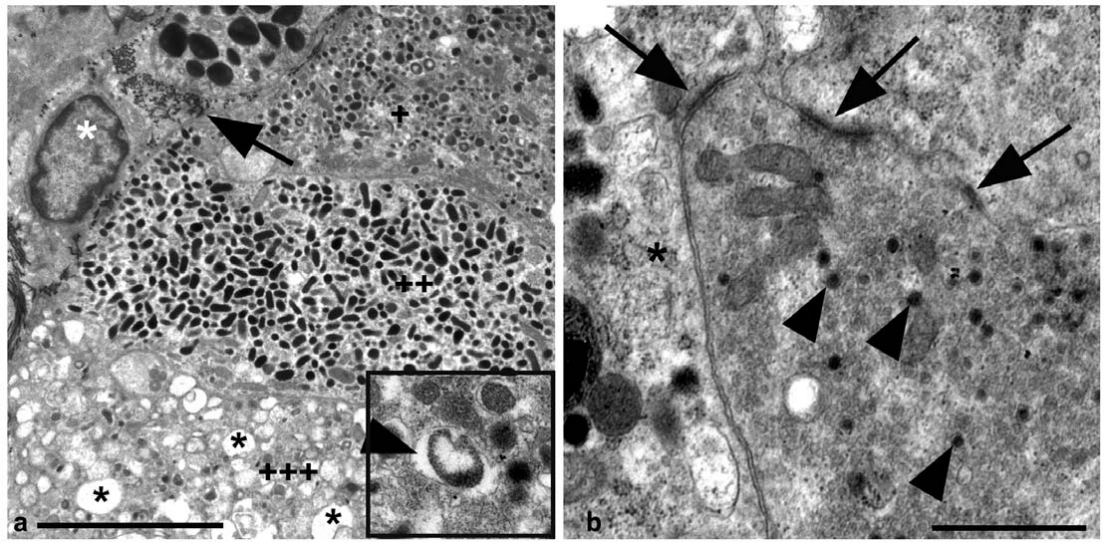
Chromaffin cell morphology. Uranyl acetate/lead citrate stain. Uninfected adrenal medulla. A: Bar = 5 µm. Three chromaffin cells can be seen, defined by multiple intracytoplasmic granules. At the top of this image (+) the chromaffin cell contains multiple moderately electron dense granules, some of which have a halo separating the contents from the granule membrane. The halo can clearly be seen in the insert (arrowhead). Below this (++) is a chromaffin cell containing ovoid electron dense granules which have no space within the granule.+++indicates a chromaffin cell with multiple discharged granules (asterisks). A tangentially sectioned blood vessel is indicated by the presence of a fibroblast (white asterisk) and collagen (arrow). The chromaffin cells abut this blood vessel. B: Bar = 1 µm. A nerve terminal containing multiple synaptic vesicles (arrowheads) lies adjacent to a chromaffin cell (asterisk). Multiple synapses can be seen (arrows).

As described for other species [32] the medulla of both normal and scrapie infected sheep adrenals consisted predominantly of chromaffin cells characterised by the presence of numerous intra-cellular membrane bound granules dispersed throughout the cytoplasm. Intracytoplasmic granules varied in both size and electron density – often electron lucent halos separated the larger and less electron dense contents from the granule membrane while no space was present within granules containing more electron dense granules (figure 3a and insert). These darker granules often formed more irregular ovoid shapes when compared with less electron dense granules. Occasional chromaffin cells with discharged granules represented by large empty membrane bound organelles were seen (figure 3a). Neurons were also abundant within the medulla. Chromaffin cells were adjacent to sinusoidal capillaries and larger blood vessels (figure 3a). Multiple nerve endings were also observed between chromaffin cells, frequently with synaptic contacts (figure 3b). Occasional macrophages with multiple intracytoplasmic lysosomes were also present within the normal adrenal medulla, as described by Coupland et al. [32].

### Chromaffin Cell Plasmalemma is Altered in Scrapie Infected Sheep

**Figure 4:**
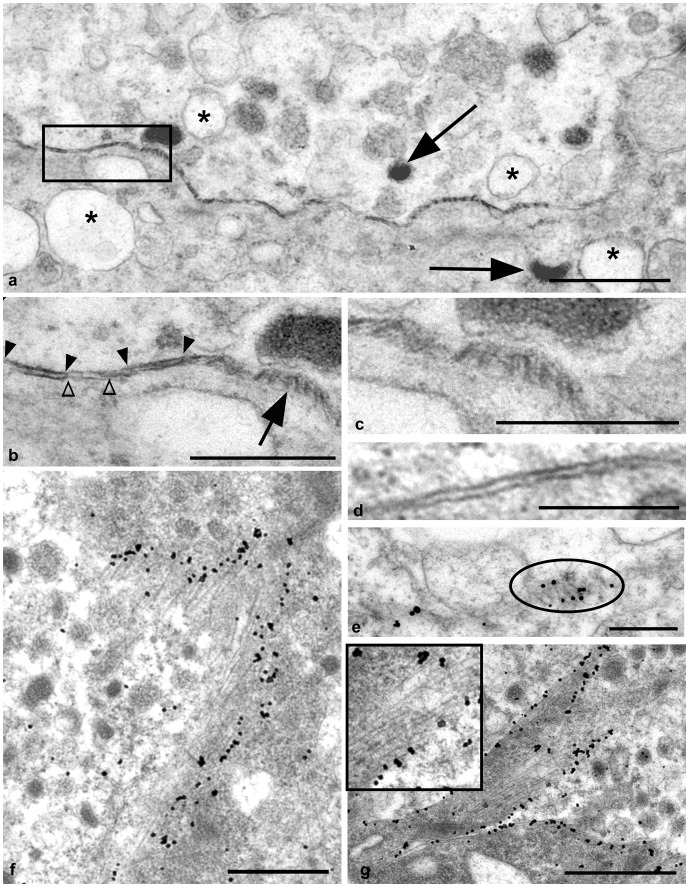
Chromaffin cell membrane abnormalities. Scrapie infected and normal adrenal medulla. A: Uranyl acetate/lead citrate counterstain. Bar = 1 µm. Scrapie infected sheep. The membrane between two chromaffin cells, viewed perpendicular to the plane of the membrane, shows periods of abnormal electron density. Both discharged (asterisk) and hormone containing granules (arrows) are present within each chromaffin cell. B: Uranyl acetate/lead citrate counterstain, Bar = 0.5 µm. Scrapie infected sheep. Higher magnification of the chromaffin cells in figure 4a (area highlighted). Electron dense membrane (between filled arrowheads) is interspersed with apparently normal membrane (between open arrowheads). Adjacent to this, the arrow indicates a section of tangentially viewed membrane contorted into regular linear palisades. C: Uranyl acetate/lead citrate counterstain, Bar = 0.3 µm. Scrapie infected sheep. Here the palisades indicated by the arrow in panel b appear more detail. D: Uranyl acetate/lead citrate counterstain, Bar = 0.3 µm. Uninfected sheep. Normal membrane between 2 chromaffin cells. E: PrP^d^ immunogold labelling. Bar = 0.4 µm. Scrapie infected sheep. Electron dense palisades form zig-zag structures within the membrane (circled). PrP^d^ labelling is limited to the polar ends of these palisades. F: PrP^d^ immunogold labelling. Bar = 0.9 µm. Scrapie infected sheep. Longer and more extended palisades, indicative of more advanced abnormality, become more erratic in organisation and lack regular spacing. Immunogold PrP^d^ labelling of these structures is abundant. G: PrP^d^ immunogold labelling. Bar = 1.5 µm. Scrapie infected sheep. Segments of abnormal membranes with developed palisades and abundant PrP^d^ accumulation frequently invaginate into the cytoplasm of chromaffin cells (indicated by multiple cytoplasmic granules). Palisades appear regular in formation (insert).

Unlike chromaffin cells of normal controls (figure 4d), segments of chromaffin cell membranes of scrapie infected sheep frequently demonstrated abnormal electron density (figure 4a) and contorted into sections of regular linear palisades of electron dense membrane. Adjacent individual palisades were usually regularly spaced with on average 30 nm between palisades, and either followed a parallel (figure 4b and in detail, 4c) or “zig–zag” formation (figure 4e). Larger and presumably more developed palisades were more frequently arranged at irregular angles (figure 4f). Often segments of abnormal membranes appeared to invaginate into the cytoplasm of the chromaffin cell (figure 4g).

### PrP^d^ Accumulation upon Chromaffin Cells Leads to Alterations in the Organisation of Chromaffin Cell Plasmalemmas

**Figure 5:**
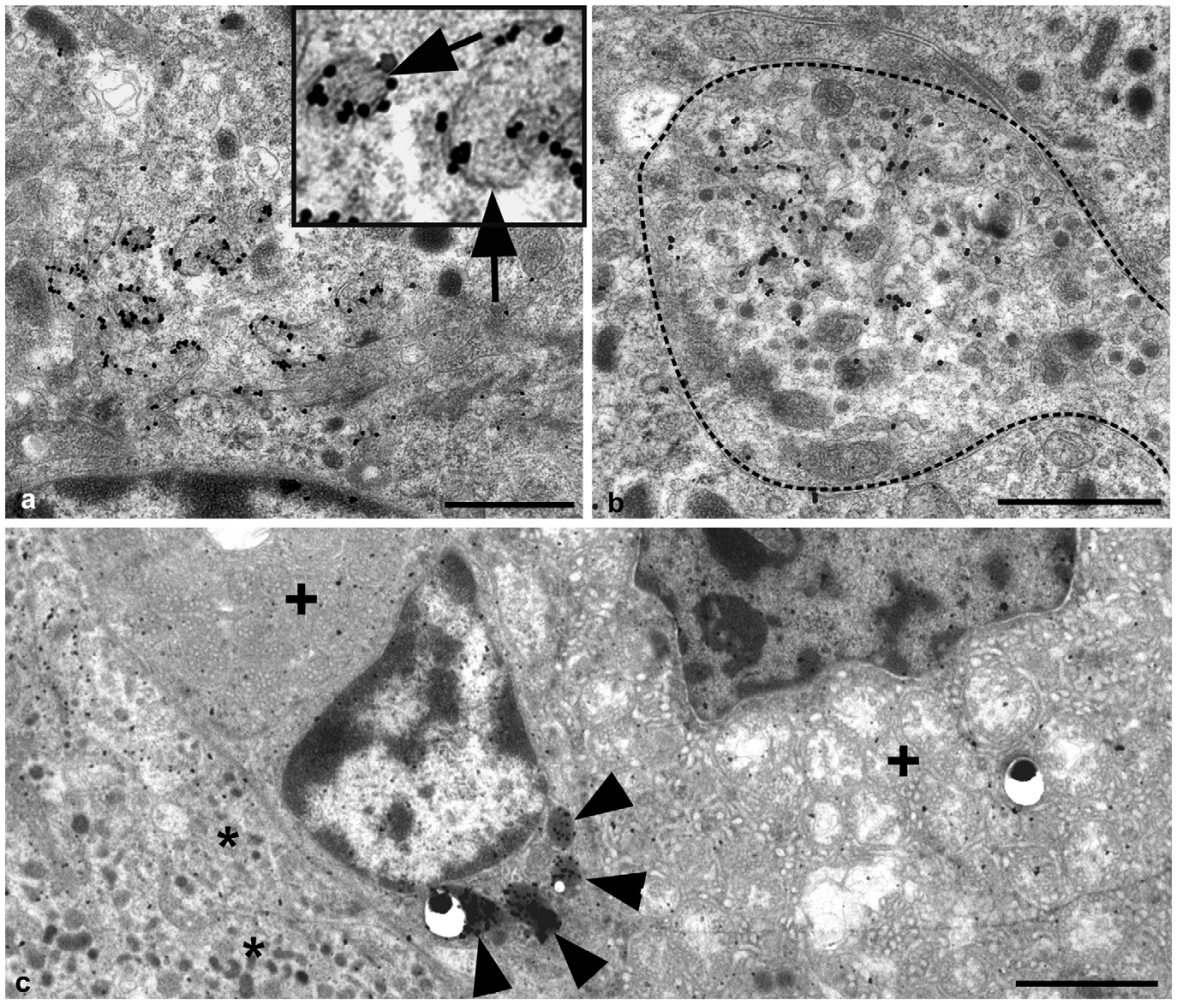
Intracytoplasmic PrP^d^ immunogold labelling of a chromaffin cell, nerve terminal and macrophage. A: Bar = 1 µm. PrP^d^ is also associated with spherical membrane profiles within the chromaffin cell, possibly chromaffin granules. Linear electron densities or palisades are present within these structures (insert and arrows). B: Bar = 1 µm. Tubular structures invaginating from the plasmalemma accumulate PrP^d^ within nerve terminals (dotted line). C: Bar = 2 µm. Intralysosomal immunogold PrP^d^ labelling (arrowheads) of a macrophage. The macrophage abuts both chromaffin cells of the adrenal medulla (asterisks), and the adrenal cortex as indicated by the presence of cells containing multiple spherical mitochondria (+).

No PrP^d^ immunolabelling was present within the adrenal medulla of any control sheep studied, however, abundant PrP^d^ was observed on or in association with the plasmalemma of chromaffin cells in scrapie affected sheep (figure 4e–g). We were unable to resolve whether membrane changes occurred predominantly on cells bearing adrenergic granules or nor-adrenergic granules. We estimated that PrP^d^ labelling was associated with more than 70% of chromaffin cells in scrapie infected adrenal medulla. While labelling was observed in association with apparently normal chromaffin cell plasmalemma which exhibited no disease specific alterations, we estimated that more than 50% of chromaffin cells which accumulated PrP^d^ also demonstrated areas of morphologically abnormal plasmalemma as described above. Membrane abnormalities were invariably associated with PrP^d^ accumulation. Labelling was restricted to the end of individual electron dense linear palisades (Figure 4e–g), suggesting that the electron dense membrane palisade comprises of molecules other than PrP^d^. Granule membranes within the chromaffin cell cytoplasm often demonstrated PrP^d^ accumulation. Linear electron dense membranes are present within these structures suggesting that the profiles were internalised from the plasmalemma of the cell, possibly following fusion of exocytic catecholamine granules (Figure 5a and insert).

Occasionally, discrete PrP^d^ accumulations were identified within nerve terminals in association with tubular structures which appeared to originate at the cell membrane (figure 5b). While this may represent transfer of PrP^d^ between peripheral nerve terminals and chromaffin cells, PrP^d^ accumulation was also found on chromaffin cells adjacent to endothelial cells surrounding blood vessels. These observations do not discriminate between possible transfer of infectivity to chromaffin cells via nerves or vascular sources. PrP^d^ was also observed within the lysosomes of infrequent macrophages, most often within the connective tissue stroma interface with the adrenal cortex (figure 5c).

## Discussion

In this study, we show that an assumption made from light microscopy, that PrP^d^ accumulated in association with nerve terminals, was misleading. While PrP^d^ was occasionally associated with nerve terminals these were insufficiently frequent to account for the magnitude of PrP^d^ observed by light microscopy. We show that accumulation of PrP^d^ is predominantly associated with abnormalities of the plasmalemmas of chromaffin cells. While we cannot precisely quantify the proportion of cells which accumulate PrP^d^, we can estimate that at least 70% of chromaffin cells visualised using our techniques accumulate PrP^d^ at the plasmalemma, and that of these, plasmalemmal malformations were seen in more than 50% of the cells studied. Membrane abnormalities associated with PrP^d^ accumulation is a feature previously described in cells of the CNS of TSE infected sheep [14], cattle [11] and experimental mouse models [12], and in the scrapie infected LRS of sheep [33] and mice [16], [34]. While the nature of the membrane defects differ according to cell type and tissue, all co-localise with the accumulation of PrP^d^. In each of the CNS studies, PrP^d^ was associated with abnormal spiral and tubular clathrin coated pit invaginations of neuronal plasma membranes, while irregular membrane folding of the plasmalemma of sheep astrocytes also co-localised with PrP^d^ accumulation [14]. We have previously suggested that in neurons, PrP^d^ interacts with a membrane-spanning protein complex which results in both excess ubiquitination and abnormal development of elongated pits. Within scrapie infected sheep, abnormal convoluted endoplasmic reticulum networks within TBMs and extended FDC dendrites both co-localise with PrP^d^ and ubiquitin further suggesting that morphological abnormalities of the plasmalemma are a common pathological effect of PrP^d^ accumulation [33]. The present study further suggests that a key toxic effect of PrP^d^ is on the integrity of membranes. As the precise nature of the morphological defect differs for different cell types this suggests that PrP^d^ interacts with different membrane molecules in different cell membranes. These observations support the concept of PrP^c^ as a scaffolding molecule involved in the assembly of multi-component signalling molecules at the cell surface [35].

Following synthesis within the endoplasmic reticulum (ER), PrP^c^ is transported via the golgi apparatus, where it is glycosylated, before reaching plasmalemma where it is primarily associated with cholesterol and sphingolipid-rich lipid rafts [36]. PrP^c^ is converted to the disease specific form at the cell plasmalemma. Here, PrP^c^ is converted to PrP^d^ following insertion of a PrP^d^ aggregate into the plasmalemma [37], which then converts normal PrP^c^ to PrP^d^ possibly with the assistance of other co-factors such as sulfated glycosaminoglycan (GAG) [38]. While this mechanism is not well defined, it is generally accepted that it occurs using a template or seeding mechanism [39]. Membranes demonstrating abundant accumulation of PrP^d^ have been shown to be unusually linear [39]. We suggest that the linear palisades observed in the present study may represent abnormal rigid sections of membranes caused by a localised PrP^d^ induced re-distribution of lipids.

Several studies have demonstrated an alteration in adreno-cortical hormones in scrapie infected animals [40], [41]. We hypothesise that the previously described changes in catecholamine levels in scrapie infected animals may be linked to the chromaffin cell membrane abnormalities we describe here. In contrast to current dogma [41], adrenal dysfunction may therefore be a result of pathological changes within the adrenal gland rather than arising from damage to the pituitary neurohypophysis.

Although changes in adreno-cortical hormones have been well reported [41]–[43], parallel investigations of adrenal medullary function in TSE affected animals are absent. Bondiolotti et al. demonstrated an alteration in sympathetic nerve function following intraperitoneal inoculation of mice with scrapie. The authors suggest that the spread of infection from the LRS to the brain resulted in a functional abnormality of the adrenal gland with a significant increase in plasma NA and subsequently blood pressure [42]. We analysed a panel of tissues including adrenal medulla, cranio-mesenteric ganglion, CNS, LRS and Peyer’s Patches, from pre-clinically affected sheep at various time points in order to determine whether this abnormality in adrenal medulla function occurs as a result of transport of infectivity and PrP^d^ from the CNS, or whether the adrenal medulla accumulates PrP^d^ following replication and amplification within the LRS and Peyer’s Patches prior to neuroinvasion (data not shown). Due to a limited number of samples available at key time points we were unable to provide a definitive conclusion regarding whether chromaffin cell infection occurs as a result of transportation of infectivity from or towards the CNS via the splanchnic nerve, or via some other means, for example the blood. The proximity of PrP^d^ positive chromaffin cells to blood vessels does however provide for the possible release of PrP^d^ and/or infectivity into the blood.
